# Causal Relationships Between Iron Deficiency Anemia, Gut Microbiota, and Metabolites: Insights from Mendelian Randomization and In Vivo Data

**DOI:** 10.3390/biomedicines13030677

**Published:** 2025-03-10

**Authors:** He Zhou, Zhenzhen Fan, Yu Da, Xiaoning Liu, Chen Wang, Tiantian Zhang, Jiaqi Zhang, Tong Wu, Jie Liang

**Affiliations:** State Key Laboratory of Holistic Integrative Management of Gastrointestinal Cancers, National Clinical Research Center for Digestive Diseases, Xijing Hospital of Digestive Diseases, Fourth Military Medical University, Xi’an 710032, China; zh505593673@163.com (H.Z.); fzhenzhen1212@163.com (Z.F.); 18093112032@163.com (Y.D.); baizhifino2022@163.com (X.L.); wangchen1059827114@outlook.com (C.W.); 13572518722@163.com (T.Z.); zjqkqjq77@163.com (J.Z.)

**Keywords:** iron deficiency anemia, gut microbiota, metabolites, mendelian randomization

## Abstract

**Background**: Iron deficiency anemia (IDA) is a common type of anemia in children and pregnant women. The effects of iron deficiency on gut microbiota and metabolic profiles are not fully understood. **Methods:** Mendelian randomization (MR) analysis was conducted to explore associations among IDA, gut microbiota, and metabolites. MR analysis was conducted using computational methods, utilizing human genetic data. Data were obtained from genome-wide association studies (GWAS), with inverse-variance-weighted (IVW) as the primary method. Animal models evaluated the effects of IDA on gut microbiota and metabolic profiles. **Results:** IVW analysis revealed significant associations between gut microbial taxa and IDA. The genus *Desulfovibrio* was protective (OR = 0.85, 95% CI: 0.77–0.93, *p* = 0.001), while *Actinomyces* (OR = 1.12, 95% CI: 1.01–1.23, *p* = 0.025) and family *XIII* (OR = 1.16, 95% CI: 1.01–1.32, *p* = 0.035) increased IDA risk. Glycine was protective (OR = 0.95, 95% CI: 0.91–0.99, *p* = 0.011), whereas medium low density lipoprotein (LDL) phospholipids increased risk (OR = 1.07, 95% CI: 1.00–1.15, *p* = 0.040). Animal models confirmed reduced *Desulfovibrio*, increased *Actinomyces*, and altered metabolites, including amino acids and phospholipids. **Conclusions:** IDA significantly impacts gut microbiota and metabolic profiles, offering insights for therapeutic strategies targeting microbiota and metabolism.

## 1. Introduction

Iron deficiency anemia (IDA) is one of the most common types of anemia worldwide, characterized by insufficient iron intake, absorption disorders, or increased demand, leading to systemic iron deficiency and impaired hemoglobin synthesis [1]. Dietary iron exists in two forms: heme iron, which is efficiently absorbed and mainly found in animal-based foods, and non-heme iron [2]. Insufficient dietary iron intake or reduced iron bioavailability is one of the leading causes of high IDA prevalence in many countries [3,4,5].

The relationship between dietary iron intake and gut microbiota has recently become a research focus. Iron is not only an essential micronutrient for host metabolism but also a critical nutrient for the growth of gut microorganisms [6]. The supply and form of dietary iron significantly influence the composition and diversity of gut microbiota. For instance, high-iron diets may promote the proliferation of certain potentially pathogenic bacteria, thereby disrupting gut microbial balance [7]. Similarly, there may be complex interactions between IDA and gut microbiota, which may play a causal role in the onset and progression of IDA [8,9]. Iron deficiency can reduce the availability of iron within the gut, altering the abundance of specific microbial populations. Iron-dependent microbes may decrease, while others may proliferate as they adapt to environmental stress. Conversely, gut microbiota can regulate the host’s iron absorption and distribution through their metabolites, such as short-chain fatty acids and bile acids, further influencing the onset and progression of IDA. In this study, we hypothesize that IDA causally affects the gut microbiota composition and metabolism and that changes in these factors subsequently influence IDA progression. Although some studies have preliminarily explored the potential effects of IDA on gut microbiota, the underlying mechanisms remain unclear. Whether this relationship is causal and the signaling pathways or metabolic networks involved require further investigation.

Blood and gut metabolites may be critical mediators linking IDA and gut microbiota. IDA disrupts host iron metabolism and causes significant changes in the levels of key blood metabolites, such as amino acids, lipids, and molecules related to energy metabolism [10]. These metabolic shifts may exert selective pressures on the growth and metabolism of gut microbes, thereby altering the composition and function of the gut microbiota [6]. Additionally, gut metabolites, including short-chain fatty acids (SCFAs), bile acids, and microbially derived secondary metabolites, are not only key mediators of host-microbiota interactions but also essential regulators of iron absorption and distribution [6,11]. For example, SCFAs are believed to influence iron absorption efficiency by modulating intestinal barrier function and inflammatory states. Bile acids and their derivatives may indirectly participate in iron metabolism by regulating bile acid receptor (e.g., FXR) signaling pathways. These complex interactions among metabolites may bridge the gap between IDA and changes in gut microbiota, offering new perspectives for understanding their underlying mechanisms. However, research on the role of blood and gut metabolites in mediating the relationship between IDA and gut microbiota remains limited. Whether these metabolites act as mediators and the specific regulatory mechanisms involved have yet to be systematically elucidated.

Mendelian randomization (MR) is a genetic variant-based method for causal inference that leverages the random distribution of genetic variations to minimize the influence of confounding factors and reverse causation commonly encountered in observational studies [12]. MR analysis offers unique advantages in studying the relationship between IDA and gut microbiota. Given that genetic and environmental factors influence the composition and function of gut microbiota, MR can eliminate environmental interference, providing robust support for identifying potential causal relationships between gut microbial traits and IDA [13]. Moreover, MR analysis allows the exploration of whether blood and gut metabolites mediate in this association, thereby unveiling underlying mechanisms.

In this study, we aim to validate the causal effects of gut microbiota on IDA using MR analysis and explore how blood and gut metabolites might mediate this relationship. This study systematically investigates the complex relationships among IDA, gut microbiota, and metabolites by integrating MR analysis with animal experiments. MR analysis validated the potential causal relationship between gut microbiota and IDA using large-scale genome-wide association studies (GWAS) data. Additionally, animal models were employed to examine the effects of IDA on gut microbial composition and metabolic features in greater detail. This research provides critical evidence for elucidating the relationship between IDA and gut microbiota and lays the groundwork for understanding its pathological mechanisms.

## 2. Materials and Methods

### 2.1. Study Design

**Figure 1:**
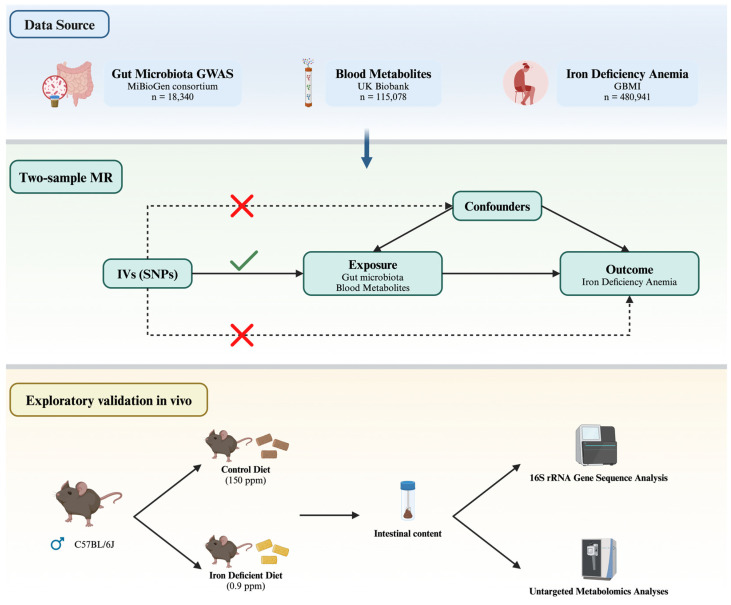
Study design integrating Mendelian randomization and in vivo validation to investigate the causal relationship between gut microbiota, blood metabolites, and iron deficiency anemia.

Figure 1 illustrates our study design, highlighting the data sources, the three assumptions underlying the causal interpretation of MR estimates, and the in vivo experimental validation. An effective genetic instrumental variable must satisfy three core assumptions: (1) Relevance assumption—the selected instrumental variable must be significantly associated with the exposure. (2) Independence assumption—the instrumental variable must not significantly correlate with potential confounders that may influence the exposure or the outcome. (3) Exclusion restriction—the instrumental variable must affect the outcome solely through the pathway of “instrumental variable → exposure → outcome”.

This study utilized publicly available data. For all individual studies within each genome-wide association study, the relevant Institutional Review Board approved GWAS, and informed consent was obtained from participants or, when necessary, from a caregiver, legal guardian, or other proxy. This study was conducted strictly following the principles outlined in the STROBE-MR Statement, which provides guidelines for reporting MR studies within observational epidemiology [13].

In this study, MR analysis based on human data is primarily used to explore the causal relationship between IDA and gut microbiota, while the mouse experiments aim to independently validate the microbial changes suggested by MR in an in vivo model rather than merging them into a causal relationship with MR.

### 2.2. Data Source and Instruments

The characteristics of the corresponding GWAS data sources are detailed in Supplementary Table S1. Gut microbiota datasets were obtained from the MiBioGen database, which includes information from 18,340 participants across 24 population-based cohorts (72.3% of European ancestry and 27.7% from Middle Eastern, East Asian, Hispanic/Latino, and African American populations). The dataset covers 211 gut microbial taxa spanning from phylum to genus levels [14]. After excluding 15 unidentified taxa, 196 known taxa (9 phyla, 16 classes, 20 orders, 32 families, and 119 genera) were retained as exposure factors. The blood metabolite data were obtained from the UK Biobank, comprising information from 115,078 European-ancestry participants. This dataset included 249 plasma measurements, covering lipids, fatty acids, and small molecules such as amino acids, ketone bodies, and glycolysis-related metabolites.

### 2.3. Selection and Verification of Instrumental Variables

Instrumental variables (IVs) were selected based on the three core assumptions for MR studies: relevance, independence, and exclusion restriction. Single nucleotide polymorphisms (SNPs) associated with gut microbiota and blood metabolites (exposure) were identified from the MiBioGen and UK Biobank database, respectively, using a significance threshold of *p* < 1.0 × 10^−5^ and a linkage disequilibrium criterion of r^2^ < 0.001 to ensure independence [15]. F-statistics were calculated for each SNP to confirm instrument strength, and only those with F-statistics > 10 were included to minimize weak instrument bias [16,17]. Heterogeneity among the selected SNPs was evaluated using Cochran’s Q test, with *p* > 0.05 indicating consistent effects across instruments. In contrast, the MR-Egger intercept test assessed horizontal pleiotropy, excluding SNPs showing evidence of pleiotropy (*p* < 0.05). A “leave-one-out” sensitivity analysis further verified that no single SNP disproportionately influenced the results.

### 2.4. MR Analysis

This study employed the inverse-variance-weighted (IVW) method as the primary approach for MR analysis to evaluate the causal relationships between gut microbiota, blood metabolites, and IDA. Complementary methods, including MR-Egger regression, weighted median, weighted mode, and simple mode, were applied to ensure the robustness of the results and to account for potential violations of core MR assumptions [18]. Causal effect estimates were expressed as odds ratios (ORs) with 95% confidence intervals (95% CIs), and statistical significance was set at α = 0.05. OR values were calculated for gut microbiota and blood metabolites to assess their causal effects on IDA. An OR greater than 1 indicated an increased risk of IDA with higher levels of exposure, while an OR less than 1 suggested a protective effect. A causal relationship was deemed significant when *p* < 0.05, and the 95% CI did not include 1.

Sensitivity analyses were conducted to validate the reliability of the MR results. Cochran’s Q test was used to assess heterogeneity (*p* > 0.05 indicating no significant heterogeneity), and the MR-Egger intercept test was applied to detect horizontal pleiotropy (*p* > 0.05). The MR-PRESSO global test was additionally performed to identify horizontal pleiotropy, with *p* > 0.05 suggesting the absence of pleiotropic effects. A leave-one-out analysis was conducted to ensure that no single SNP disproportionately influenced the results. Visual inspections, including funnel and scatter plots, were used to evaluate the consistency and robustness of the causal estimates.

### 2.5. Animals

Male SPF C57BL/6 mice, aged 8 weeks and weighing 20 ± 2 g, were provided by the Animal Care and Use Committee of the Air Force Medical University. All experimental procedures were approved by the Ethics Committee for Animal Experiments (Approval No. 20231013) and conducted following the National Institutes of Health (NIH) guidelines. The control group (Ctrl) received a standard chow diet (D08080401; Research Diets, NJ, USA), while the IDA groups were fed low-iron diets (D08080402; Research Diets). All diets were formulated based on the AIN-76A standard [19]. Before this study, mice were acclimated to the experimental diet and housing conditions for five days. Subsequently, they were maintained on their respective diets for 60 days, during which their weight and general health were monitored daily.

### 2.6. DNA Extraction and 16S rRNA Gene Sequence Analysis

Microbial DNA was extracted from intestinal contents using the PF Mag-Bind Stool DNA Kit (Omega Bio-tek, Norcross, GA, USA), following the manufacturer’s protocol. The quality and concentration of the extracted DNA were evaluated via agarose gel electrophoresis and a NanoDrop^®^ ND-2000 spectrophotometer (Thermo, Waltham, MA, USA). The V3-V4 hypervariable regions of the 16S rRNA gene were amplified using primers 338F (5′-ACTCCTACGGGAGGCAGCAG-3′) and 806R (5′-GGACTACHVGGGTWTCTAAT-3′) on an ABI GeneAmp^®^ 9700 PCR thermocycler (Applied Biosystems, Foster City, CA, USA). Each PCR reaction was performed in triplicate to ensure reproducibility. The amplified products were purified and quantified with a Quantus^™^ fluorometer (Promega, Madison, WI, USA). The purified amplicons were pooled and sequenced using the Illumina PE300/PE250 platform (Illumina, San Diego, CA, USA), following the standard protocols provided by Majorbio Bio-Pharm Technology Co. Ltd. (Shanghai, China). Raw sequencing data, deposited in the NCBI SRA database (Accession: SRP552378), were processed into operational taxonomic units (OTUs) at 97% similarity. Downstream analyses were performed using QIIME (v1.7.0) to calculate weighted UniFrac distances. Statistical comparisons of bacterial taxa were carried out using the Student’s *t*-test, and differences in taxa among samples were further explored using Linear Discriminant Analysis Effect Size (LEfSe).

### 2.7. Untargeted Metabolomics Analyses

To extract metabolites, 5 mg of cecal content was placed in a 2 mL tube containing a 6 mm grinding bead and 400 μL of extraction solution (methanol: water = 4:1, *v*:*v*) spiked with 0.02 mg/mL L-2-chlorophenylalanine as an internal standard. The samples were processed using grinding at −10 °C, sonication at 5 °C, and subsequent centrifugation. The resulting supernatant was collected for LC-MS/MS analysis, conducted on a Thermo UHPLC-Q Exactive HF-X system equipped with an ACQUITY HSS T3 column, operated at a 0.4 mL/min flow rate. Raw LC-MS data were preprocessed using Progenesis QI software (version 2.4; Waters Corp., Milford, MA, USA), and metabolite identification was achieved by querying databases such as the Human Metabolome Database (HMDB, http://www.hmdb.ca/, accessed on 1 October 2023).

### 2.8. Statistical Analysis

All statistical analyses were performed using R software (version 4.3.1), and specific MR analyses utilized the TwoSampleMR package (version 0.6.4). For MR analyses, odds ratios (ORs) and 95% confidence intervals (95% CIs) were calculated to estimate gut microbiota’s and blood metabolites’ causal effects on IDA. Statistical significance was defined as *p* < 0.05, and multiple testing corrections were applied using the Bonferroni method to control for type I errors. Differences between experimental groups were analyzed using the Student’s *t*-test for normally distributed data. Correlations between gut microbiota, blood metabolites, and iron metabolism markers were analyzed using Spearman’s correlation coefficient.

## 3. Results

### 3.1. Instrument Variables Included

After conducting a series of quality control measures for IDA, we extracted 29 independent SNPs (*p* < 1.0 × 10^−5^, r^2^ < 0.001) that were associated with one bacterial family and two bacterial genera. These SNPs were selected as IVs based on their significant genetic association with gut microbiota. All selected IVs exhibited F-statistics greater than 10, indicating sufficiently strong IV effects and minimal risk of weak IV bias. Detailed results of IV association between gut microbiota and IDA can be found in Supplementary Table S2.

Cochran’s Q test for the IVW method revealed no significant heterogeneity among the gene IVs related to IDA (*p* > 0.05), as shown in Supplementary Table S4. This suggests that the effect of the gene IVs on IDA is consistent across the selected SNPs. Additionally, the MR-Egger intercept test revealed no significant pleiotropy (*p* > 0.05), indicating that the gene IVs are not influenced by alternative pathways other than the hypothesized relationship between gut microbiota and IDA (Supplementary Table S5). Therefore, all selected gene IVs can be considered valid IVs for this MR analysis of gut microbiota’s impact on IDA.

### 3.2. MR Analysis of Gut Microbiota’s Effect on IDA

**Figure 2:**
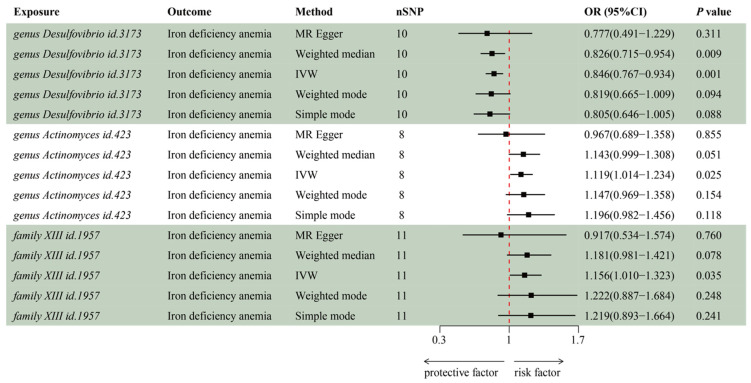
Forrest plot for the summary causal effects of gut microbiota on iron deficiency anemia risk based on MR methods (MR Egger, Weighted median, IVW, Weighted mode, Simple mode). The red dashed vertical line represents the null effect (OR = 1.0). The microbiota data are derived from the MiBioGen database, which includes a total of 18,340 participants. The summary data for IDA in the GBMI consists of 480,941 participants (468,624 cases and 2317 controls). SNP, single nucleotide polymorphisms; OR, odds ratio; IVW, inverse-variance-weighted.

**Figure 3:**
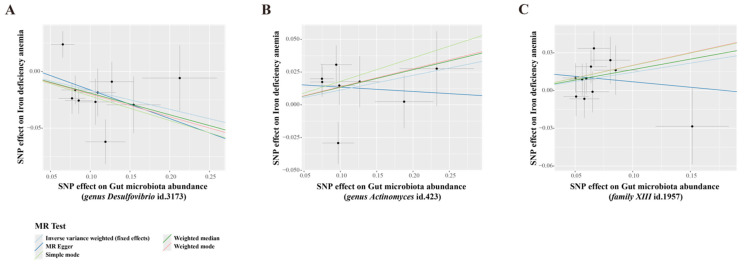
Scatter plots for the causal effects of gut microbiota on iron deficiency anemia risk based on MR methods (MR Egger, Weighted median, IVW, Weighted mode, Simple mode). (**A**) genus *Desulfovibrio* (ID: 3173). (**B**) genus *Actinomyces* (ID: 423). (**C**) family *XIII* (ID: 1957). The microbiota data are derived from the MiBioGen database, which includes a total of 18,340 participants. The summary data for IDA in the GBMI consists of 480,941 participants (468,624 cases and 2317 controls). SNP, single nucleotide polymorphisms. Each dot represents an individual SNP, with the x-axis indicating the SNP’s effect on gut microbiota abundance and the y-axis indicating the SNP’s effect on iron deficiency anemia.

**Figure 4:**
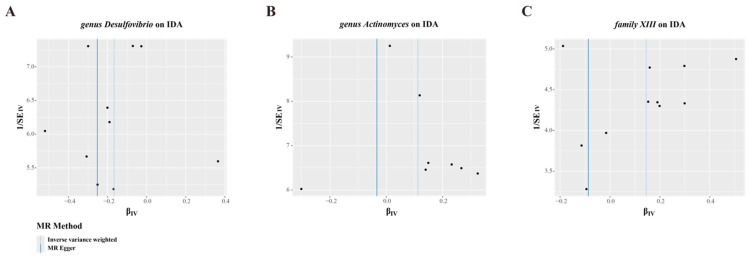
Funnel plots of causal estimates from genetically predicted microbiota on iron deficiency anemia. (**A**) genus *Desulfovibrio* (ID: 3173). (**B**) genus *Actinomyces* (ID: 423). (**C**) family *XIII* (ID: 1957). The microbiota data are derived from the MiBioGen database, which includes a total of 18,340 participants. The summary data for IDA in the GBMI consists of 480,941 participants (468,624 cases and 2317 controls). Each dot represents an individual SNP’s effect estimate, with the x-axis indicating the effect size (β_IV) and the y-axis indicating the precision (1/SE).

**Figure 5:**
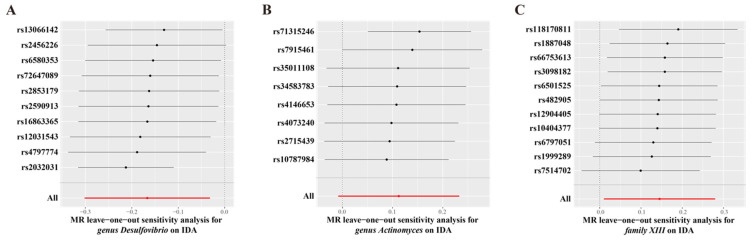
Leave-one-out plots of causal estimates from genetically predicted microbiota on iron deficiency anemia. (**A**) genus *Desulfovibrio* (ID: 3173). (**B**) genus *Actinomyces* (ID: 423). (**C)** family *XIII* (ID: 1957). The microbiota data are derived from the MiBioGen database, which includes a total of 18,340 participants. The summary data for IDA in the GBMI consists of 480,941 participants (468,624 cases and 2317 controls). The red line indicates the overall effect estimate when all SNPs are included.

IVW estimation showed that genus *Desulfovibrio* (ID: 3173) had a protective effect on IDA (OR = 0.85, 95% CI: 0.77–0.93, *p* = 0.001), suggesting a reduced risk of IDA with higher levels of this genus. In contrast, genus *Actinomyces* (ID: 423; OR = 1.12, 95% CI: 1.01–1.23, *p* = 0.025) and family *XIII* (ID: 1957; OR = 1.16, 95% CI: 1.01–1.32, *p* = 0.035) were associated with an increased risk of IDA, indicating their role as pathogens or opportunistic pathogens in the development of IDA (Figure 2, Supplementary Table S3). The MR-Egger and scatter plot demonstrated no horizontal pleiotropy or outliers (*p* > 0.05, Figure 3). Cochrane’s Q test and funnel plot indicated no significant heterogeneity among the selected SNPs (*p* > 0.05, Figure 4). The reverse MR analysis showed no evidence of a causal relationship between IDA and the gut microbiota (Supplementary Table S6). The “MR Leave-one-out” sensitivity analyses demonstrated that removing individual SNPs did not alter the overall causal inference results (Figure 5). This indicates that no single IV was solely driving the observed causal associations.

### 3.3. MR Analysis of Blood Metabolites’ Effect on IDA and Gut Microbiota

The IVW estimation revealed that glycine had a protective effect on IDA (OR = 0.95, 95% CI: 0.91–0.99, *p* = 0.011, Table 1). This indicates that higher glycine levels are associated with a reduced risk of IDA. Conversely, phospholipids in medium low density lipoprotein (LDL) were associated with an increased risk of IDA (OR = 1.07, 95% CI: 1.00–1.15, *p* = 0.040, Table 1), suggesting that elevated levels of these phospholipids may contribute to the development of IDA. MR-Egger test indicated no horizontal pleiotropy (*p* > 0.05, Supplementary Table S7). Cochran’s Q test for the IVW method revealed no significant heterogeneity among the gene IVs related to IDA (*p* > 0.05, Supplementary Table S7). However, the IVW estimation also demonstrated no significant association between blood metabolites and the identified gut microbiota, indicating that the observed effects of blood metabolites on IDA are likely independent of gut microbiota (Supplementary Table S8).

### 3.4. Iron Deficiency Anemia Affects Gut Microbiota Composition and Diversity

**Figure 6:**
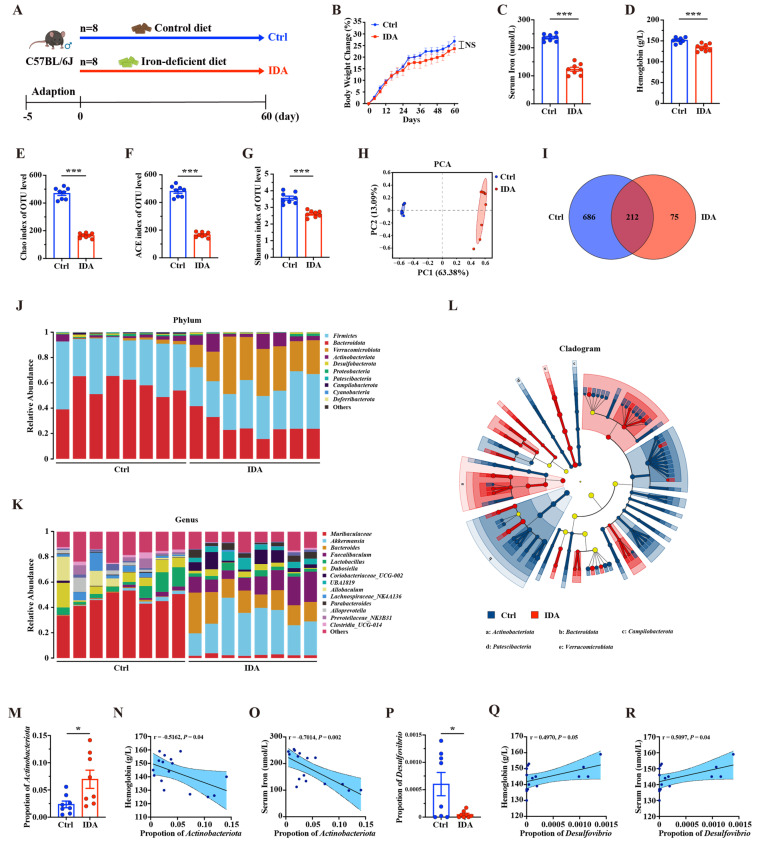
Effects of iron-deficient diet-induced iron deficiency anemia on mice’s body weight, iron metabolism, and gut microbiota composition. (**A**) Schematic of the experimental design. (**B**) Body weight changes. (**C**) Serum iron levels. (**D**) Hemoglobin concentrations. (**E**) Chao index of gut microbiota. (**F**) ACE index of gut microbiota. (**G**) Shannon index of gut microbiota. (**H**) Principal component analysis (PCA) of gut microbiota composition. (**I**) Venn diagram of shared OTUs between groups. (**J**) Gut microbiota composition at the phylum level. (**K**) Gut microbiota composition at the genus level. (**L**) Cladogram from LEfSe analysis. (**M**) Relative abundance of Actinobacteriota. (**N**) Correlation between Actinobacteriota abundance and hemoglobin levels. (**O**) Correlation between Actinobacteriota abundance and serum iron levels. (**P**) Relative abundance of Desulfovibrio. (**Q**) Correlation between Desulfovibrio abundance and hemoglobin levels. (**R**) Correlation between Desulfovibrio abundance and serum iron levels. IDA, iron deficiency anemia; PCA, principal component analysis. Data represent mean ± SD. * *p* < 0.05, *** *p* < 0.001, NS = not significant. Statistical differences were analyzed using Student’s *t*-test for comparisons between two groups. PERMANOVA was applied for PCA analysis. LEfSe analysis was used to identify differentially abundant taxa. Correlation analyses were performed using Spearman’s rank correlation. *n* = 8 mice/group.

To investigate the changes in the gut microbiota of IDA, a mouse model of IDA was established by feeding the IDA group a low-iron diet for 60 days (Figure 6A). The results showed no significant difference in body weight between the IDA and control groups during the modeling period (Figure 6B). However, on day 60, serum iron levels and hemoglobin concentrations in the IDA group were significantly lower than those in the control group, confirming the successful establishment of the IDA model (Figure 6C,D). Further analysis revealed significant differences in gut microbiota diversity and composition between the IDA and control groups. The α-diversity analysis with Chao, ACE, and the Shannon index showed a substantial reduction in gut microbiota richness and diversity in the IDA group (Figure 6E–G). Principal component analysis (PCA) based on UniFrac distances showed a distinct clustering of microbiota composition in the IDA group compared to the control group (Figure 6H). Commonality analysis also revealed significantly fewer shared bacterial taxa in the IDA group (Figure 6I). At the phylum level, the IDA group showed a significant decrease in the relative abundance of *Bacteroidota* and an increase in *Verrucomicrobiota* and *Actinobacteriota* compared to the control group (Figure 6J,M). At the genus level, the relative abundance of *Desulfovibrio* was significantly reduced in the IDA group (Figure 6K,P). Linear discriminant analysis (LDA) and LEfSe identified key microbial taxa enriched in the control group (*Bacteroidota, Campylobacterota,* and *Patescibacteria*) and the IDA group (*Actinobacteriota* and *Verrucomicrobiota*) (Figure 6L). Correlation analyses were performed to explore the relationship between these microbes and IDA between the relative abundance of *Actinobacteriota* and *Desulfovibrio* and serum iron and hemoglobin levels. The relative abundance of *Actinobacteriota* showed a significant negative correlation with serum iron and hemoglobin levels, suggesting that an increase in *Actinobacteriota* is associated with decreased iron and hemoglobin levels (Figure 6N,O). Conversely, the relative abundance of *Desulfovibrio* showed a significant positive correlation with serum iron and hemoglobin levels (Figure 6Q,R). In conclusion, this study demonstrated that IDA significantly affects mice’s gut microbiota composition and diversity. Notably, the relative abundance of *Actinobacteriota* and *Desulfovibrio* has changed significantly in IDA.

### 3.5. Iron Deficiency Anemia Impacts Gut Microbial Metabolites

**Figure 7:**
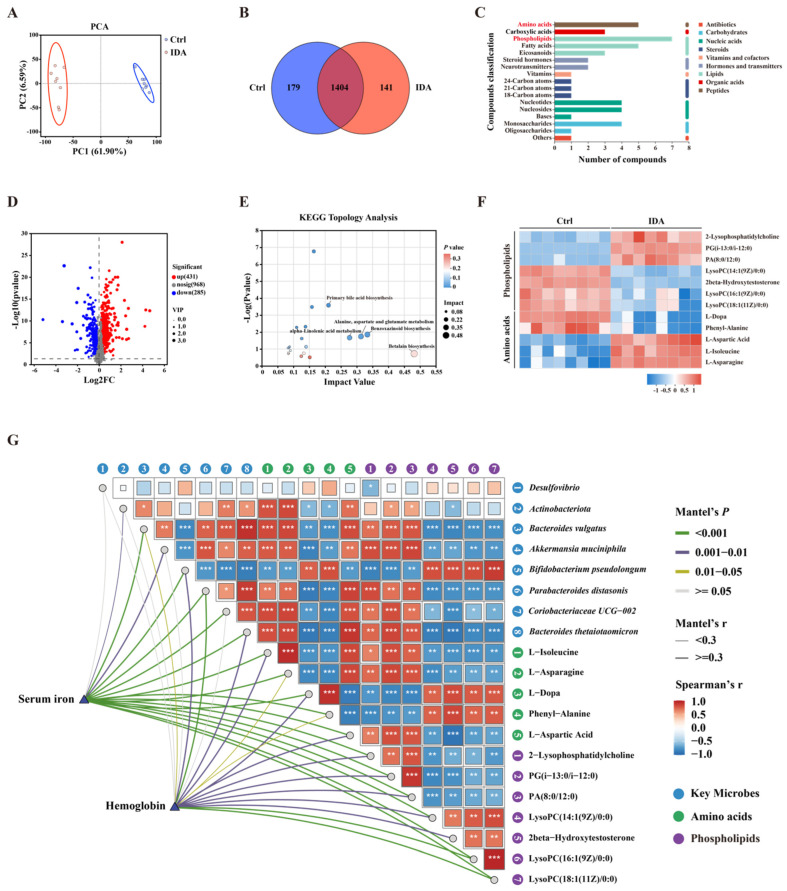
Iron deficiency anemia significantly affects gut microbial metabolites, particularly amino acids and phospholipids. (**A**) Principal components analysis (PCA) of metabolomic profiles. (**B**) Venn diagram of shared and unique metabolites. (**C**) Classification of metabolites into functional categories. (**D**) Volcano plot of differentially abundant metabolites. Red dots represent significantly upregulated metabolites, blue dots represent significantly downregulated metabolites, and gray dots represent metabolites with no significant changes. (**E**) KEGG pathway topology analysis of impacted pathways. Bubble size represents pathway impact, and bubble color intensity reflects statistical significance (darker is more significant). (**F**) Heatmap of the relative abundances of key metabolites (amino acids and phospholipids). Red indicates higher abundance, and blue indicates lower abundance. (**G**) Pairwise correlations of serum iron, hemoglobin, gut microbiota, and metabolites. The heatmap shows the results of the Spearman’s correlation analysis of gut microbiota and microbiota. In the triangular section of the heatmap, the gradient color represents the Spearman‘s correlation coefficient between microorganisms and metabolites. Red indicates a negative correlation, blue indicates a positive correlation, and deeper colors (larger rectangular areas) represent higher absolute correlation coefficient values. Asterisks indicate significance; * *p* < 0.05, ** *p* < 0.01, and *** *p* < 0.001. In the network, lines show the Mantel test results, displaying only correlations with r ≥ 0.3 and *p* < 0.01. IDA, iron deficiency anemia; PCA, principal component analysis. PERMANOVA was used for PCA analysis to evaluate group clustering. The students’ *t*-test was applied to compare metabolite levels between groups. Volcano plot results were based on fold change (log2FC) and −log10 (*p*-value). KEGG pathway enrichment was evaluated using Fisher’s exact test. One-way ANOVA with multiple comparisons was used for heatmap data. *n* = 8 mice/group.

To further explore the impact of IDA on gut microbial metabolites, a metabolomic profiling of intestinal contents was performed for both groups. PCA revealed that the IDA group formed a distinct cluster, indicating significant metabolic differences from the control group (Figure 7A). Venn diagram analysis showed 1404 shared metabolites between the two groups, with 141 metabolites unique to the IDA group (Figure 7B). Among the metabolites, amino acids and phospholipids were the most represented classes, consistent with the MR analysis results (Figure 7C). A total of 1684 metabolites were identified, with 431 significantly upregulated and 285 downregulated in the IDA group, as visualized in a volcano plot (Figure 7D). KEGG pathway topology analysis identified key impacted pathways, including primary bile acid biosynthesis, alanine, aspartate and glutamate metabolism, and alpha-linolenic acid metabolism (Figure 7E). Further analysis of amino acids and phospholipids revealed significant changes. Among phospholipids, 2-Lyso-phosphatidylcholine, PG(i-13:0/i-12:0), and PA(8:0/12:0) were markedly upregulated, while LysoPC species and 2beta-Hydroxytestosterone were significantly downregulated in the IDA group (Figure 7F). Among amino acids, L-Aspartic Acid, L-Asparagine, and L-Isoleucine showed relative downregulation, with L-Dopa and Phenylalanine significantly decreased (Figure 7F).

Comprehensive correlation analysis was conducted to examine interactions among serum iron, hemoglobin levels, intestinal microbes, and metabolites (Figure 7G). The Mantel test revealed significant correlations between serum iron and microbial composition (Mantel’s r > 0.3, *p* < 0.001) and between hemoglobin and metabolites. Key microbial genera, including *Desulfovibrio*, *Akkermansia muciniphila*, and *Bacteroides vulgatus*, showed strong positive or negative correlations with serum iron and hemoglobin. Among metabolites, amino acids such as L-Asparagine, L-Aspartic Acid, and Phenylalanine were positively correlated with hemoglobin levels, while phospholipids such as LysoPC(16:1(9Z)/0:0) and PA(8:0/12:0) showed strong associations with serum iron. Notably, LysoPC(18:1(11Z)/0:0) exhibited the highest positive correlation with serum iron (Spearman’s r > 0.5, *p* < 0.001). Further analysis revealed strong relationships between microbial genera and metabolites. *Desulfovibrio* was positively correlated with LysoPC(16:1(9Z)/0:0) and PA(8:0/12:0), suggesting its potential role in influencing phospholipid metabolism. Similarly, *Akkermansia muciniphila* was positively associated with L-Asparagine and L-Dopa, indicating its involvement in amino acid metabolism. Conversely, *Bacteroides vulgatus* exhibited a negative correlation with L-aspartic acid but was positively correlated with LysoPC(14:1(9Z)/0:0), suggesting opposing roles in amino acid and phospholipid metabolism.

These findings suggest that IDA profoundly alters the gut microbial composition and metabolite profiles, with specific amino acids and phospholipids playing pivotal roles in the metabolic adaptations associated with iron deficiency.

## 4. Discussion

IDA is a prevalent condition characterized by complex interactions involving gut microbiota and metabolites [20,21]. Iron deficiency reduces iron availability in the gut, leading to alterations in microbial diversity and composition [8,9]. Dysregulated iron metabolism may result in gut microbiota imbalances, which, through metabolites such as short-chain fatty acids and bile acids, influence iron absorption and inflammatory responses [6,10,11]. Recent studies have highlighted that SCFAs, particularly propionate produced by synergistic microbial interactions, may enhance intestinal barrier function and systemic iron metabolism [22]. However, the precise mechanisms linking IDA, gut microbiota, and metabolites remain unclear, and the causal relationships have yet to be fully established. This study employed MR analysis and animal models to investigate the effects of IDA on gut microbiota and metabolites. Our findings revealed that IDA significantly alters gut microbiota composition and profoundly impacts the levels of blood and gut metabolites. Unlike observational studies [10], this approach provides robust causal evidence for the regulatory effects of IDA on gut microbiota and metabolic profiles. These results not only enhance our understanding of the biological mechanisms underlying the interplay between IDA and gut microbiota but also underscore the pivotal role of metabolites in mediating host-microbiota interactions. The insights gained suggest feasible interventions, such as Desulfovibrio-targeted probiotics or glycine supplementation, to mitigate IDA progression, similar to probiotic strategies shown to improve metabolic dysregulation in obesity and diabetes [23,24].

The MR analysis revealed significant associations between specific gut microbiota, metabolites, and IDA. Notably, our findings bridge a critical gap in the literature; while observational studies hypothesized a protective role of *Desulfovibrio* in iron metabolism [25], our MR analysis provides the first genetic evidence supporting its causal protective effect against IDA. *Desulfovibrio* demonstrated a protective effect against IDA, while *Actinomyces* and Family *XIII* were associated with an increased risk of its development. Among metabolites, glycine exhibited protective effects, whereas phospholipids in LDL were linked to a higher risk of IDA. Mechanistically, *Desulfovibrio*-derived hydrogen sulfide may enhance iron absorption by stabilizing hypoxia-inducible factor 2α (HIF-2α) in enterocytes, thereby upregulating ferroportin expression [26,27,28]. Conversely, Actinomyces-induced interleukin-6 (IL-6) secretion may activate the JAK2/STAT3 pathway, increasing hepcidin production and systemic iron sequestration, a mechanism analogous to gut microbiota-mediated inflammatory pathways observed in colorectal cancer progression [29]. Glycine, known for its anti-inflammatory and metabolic-stabilizing properties, may help alleviate chronic inflammation and metabolic disturbances associated with IDA [30,31]. On the other hand, phospholipids in LDL might exacerbate iron deficiency by affecting iron transport or storage pathways [32].

The in vivo animal model further demonstrated the significant effects of IDA on gut microbiota and metabolites. However, while the animal model corroborates MR findings, its translational relevance may be constrained by interspecies differences in microbiota- host interactions. For example, murine-specific responses to iron deficiency, such as altered bile acid conjugation patterns, may not fully recapitulate human physiology, a limitation also noted in studies on gut microbial enterotypes and metabolic interventions [23,33]. Mice fed a low-iron diet exhibited substantial alterations in gut microbiota composition and diversity. Specifically, the abundance of *Desulfovibrio* decreased significantly, while *Actinomyces* increased, which is consistent with findings from the MR analysis and supports their potential roles in IDA. Additionally, reshaping the overall gut microbial structure suggests that iron deficiency may exacerbate microbial imbalance by altering interspecies interactions. Metabolite analysis revealed that IDA affected the overall levels of amino acids and phospholipids and altered their specific compositions. For instance, the levels of amino acids such as leucine and phenylalanine, which are critical for energy metabolism and protein synthesis, were significantly reduced, potentially reflecting metabolic dysregulation under iron-deficient conditions [34,35]. Among phospholipids, lysoPCs and lysoPEs associated with LDL showed significant changes [36,37]. Compared to the MR analysis, the animal model provided further insights into the changes in gut microbiota and metabolites in IDA mice under controlled experimental conditions. However, it is important to note that while the animal model can identify microbial taxa and metabolites associated with IDA, it cannot establish causal relationships between these factors and IDA. The animal model only suggests associations, whereas MR analysis provides causal inference based on human data. This distinction is crucial, as the two methods serve different purposes in the study of IDA. Importantly, MR sensitivity analyses indicated minimal horizontal pleiotropy, reinforcing the validity of our causal inferences.

This study, utilizing MR analysis and animal experiments, revealed distinct metabolic changes from different sources. MR analysis focused on blood metabolites, reflecting systemic metabolic changes, while animal experiments examined metabolites in mouse gut contents, highlighting localized intestinal metabolic activity. These two approaches target different levels of metabolism; blood metabolites capture the integrated metabolic outcomes of multiorgan interactions, such as those related to iron metabolism, inflammation, and energy balance, while gut metabolites are directly influenced by microbial activity in the local intestinal environment, offering insights into host-microbe interactions. Compared to previous studies, this research offers a novel perspective. Earlier investigations on IDA predominantly focused on changes in blood iron-related markers, such as ferritin, transferrin, and serum iron, and their associations with systemic inflammation and immune dysfunction [6,8,38,39]. However, these studies were limited to descriptive analyses of blood metabolites without exploring the role of gut microbiota-derived metabolites in systemic metabolic regulation. Additionally, while some observational studies suggested that IDA could lead to gut microbiota imbalances, they did not systematically understand the connections between gut metabolites and systemic metabolic networks [6,8]. For the first time, this study combined MR analysis and animal models to comprehensively examine both blood and gut metabolites, providing a multilayered perspective on IDA metabolism. Despite their different origins, the two types of metabolites were complementary in their mechanisms. Consistent with the systemic changes observed in blood metabolites, significant alterations in amino acids and phospholipids were also evident in gut contents. This suggests that IDA may influence systemic metabolic states through localized microbial metabolism. For instance, this study identified lysoPCs as potential key mediators linking local gut metabolism to systemic metabolic regulation. This finding bridges the gap in understanding how gut-derived metabolites influence systemic metabolic processes, which was largely unexplored in previous studies [40,41]. However, the differences between the two sources of metabolomics data also pose certain limitations. Blood metabolites reflect systemic metabolic outcomes, making directly attributing changes to specific local gut contributions challenging. Conversely, gut metabolites reveal distinct microbial metabolic changes but do not clarify their dissemination and roles in systemic metabolism. Future studies should aim to explore the connections between blood and gut metabolites through approaches such as multi-omics integration or tracer experiments with labeled metabolites. Future therapeutic strategies could combine iron supplementation with microbiota-modulating approaches, such as administering *Desulfovibrio* probiotics or dietary interventions to elevate glycine levels, as demonstrated in studies targeting metabolic syndrome through microbial metabolite modulation [24].

## 5. Conclusions

This study combined MR analysis and animal experiments to reveal the complex relationships between IDA, gut microbiota, and metabolites. MR analysis identified key microbial taxa (genus *Desulfovibrio*, genus *Actinomyces*, and family *XIII*) and blood metabolites (glycine, phospholipids in medium LDL) associated with IDA. At the same time, animal models confirmed significant changes in gut microbial composition and specific metabolites, although they only demonstrated associations, not causality. These findings provide new insights into the interplay between gut microbiota and systemic iron metabolism, offering a foundation for targeted microbiota-based therapeutic strategies for IDA.

## Figures and Tables

**Table 1:** MR estimates for the association between blood metabolites and IDA.

Exposure	Odd Ratio	95% CI	*p* Value
Glycine			
MR Egger	0.96	0.91–1.01	0.161
Weighted median	0.95	0.91–1.00	0.038
IVW	0.95	0.91–0.99	0.011
Weighted mode	0.96	0.92–1.00	0.048
Simple mode	0.90	0.75–1.09	0.301
Phospholipids in medium LDL			
MR Egger	1.12	0.99–1.26	0.087
Weighted median	1.11	0.99–1.23	0.063
IVW	1.07	1.00–1.15	0.040
Weighted mode	1.14	1.02–1.26	0.024
Simple mode	0.97	0.81–1.16	0.730

IDA, iron deficiency anemia; CI, confidence interval; IVW, inverse-variance-weighted; MR, mendelian randomization; LDL, low density lipoprotein.

## Data Availability

The original contributions presented in this study are included in the article and Supplementary Material. Further inquiries can be directed to the corresponding authors.

## Supplementary Materials

The following supporting information can be downloaded at https://www.mdpi.com/article/10.3390/biomedicines13030677/s1: Table S1: Characteristics of summary genome-wide association studies; Table S2: Instrumental variables used in the MR analysis of the association between gut microbiota and iron deficiency anemia. Table S3: Full result of MR estimates for the association between gut microbiota and iron deficiency anemia. Table S4: The heterogeneity results from Cochran’s Q test. Table S5: Pleiotropy results from Egger intercept analysis. Table S6: Reverse causal association between IDA and gut microbiota. Table S7: The heterogeneity results from Cochran’s Q test. Table S8: MR estimates for the association between blood metabolites and gut microbiota.
